# Initial experience with continuous intra-arterial fluorescent glucose monitoring in patients in the ICU following cardiac surgery

**DOI:** 10.1186/cc10781

**Published:** 2012-03-20

**Authors:** S Bird, L Macken, O Flower, E Yarad, F Bass, N Hammond, D LaCour, P Strasma, S Finfer

**Affiliations:** 1 Royal North Shore Hospital, St Leonards, NSW, Australia; 2 GluMetrics, Inc., Irvine, CA, USA

## Introduction

Continuous glucose monitoring (CGM) in ICUs has the potential to improve patient safety and outcomes. The GluCath Intravascular CGM System uses a novel quenched chemical fluorescence sensing mechanism to measure glucose concentration (BG) in venous or arterial blood. This is the first report of its use in cardiac surgery patients.

## Methods

This ongoing clinical study is evaluating the system deployed via a standard 20G radial artery catheter inserted for routine care in 20 patients undergoing cardiac surgery. Data are presented from five run-in patients. Outcome measures are qualitative (ease-of-use, workflow fit) and quantitative (accuracy vs. reference analyzer). Sensors were inserted shortly after ICU admission with placement confirmed by ultrasound and *in vivo *calibration 30 minutes later. Clinical staff managed blood glucose according to usual protocols. Glucose values were recorded each minute for 24 hours; hourly reference samples from the same arterial catheter were analyzed on a Radiometer ABL Blood Gas Analyzer.

## Results

**Figure 1:**
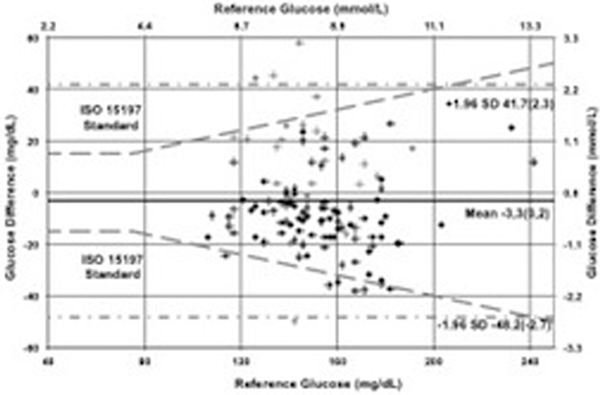
**ISO-modifi ed Bland-Altman plot**.

The sensor was successfully deployed in all five patients and did not interfere with clinical care, blood pressure monitoring or sampling. One patient suffered a cardiopulmonary arrest; the sensor functioned successfully during resuscitation and urgent return to the operating room. One hundred and twenty reference samples ranging from 5.9 to 13.4 mmol/l were collected; 107/120 (89.2%) of GluCath measurements met ISO 15197 criteria (within ±20% of reference when BG >4.2 mmol/l; Figure 1). In Subject 1 the sensor was inadvertently retracted into the arterial catheter during the study, leading to measurement error from arterial flush solution contamination. In a sensitivity analysis excluding this patient, 89/95 (93.7%) of measurements met ISO 15197 with a mean absolute relative difference of 9.4%.

## Conclusion

The GluCath System measured glucose concentration continuously in a cardiac surgery ICU without compromising arterial line function or patient care. In all patients the sensor operated without interruption for 24 hours following a single *in vivo *calibration.

